# Supplementary material for: Local DNA shape is a general principle of transcription factor binding specificity in Arabidopsis thaliana
Source: Nat Commun. 2021 Nov 12;12:6549. doi: 10.1038/s41467-021-26819-2 (PMC8590021; doi:10.1038/s41467-021-26819-2)
Supplement: Supplementary file 1 — Supplementary information. [file 41467_2021_26819_MOESM1_ESM.pdf]

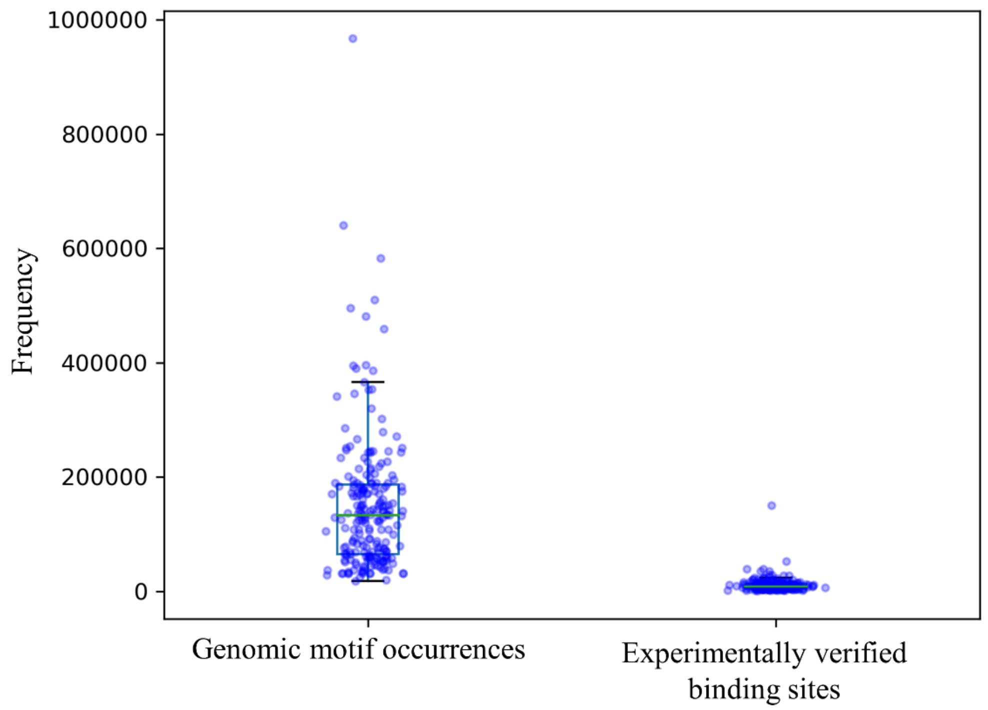

Supplementary Figure 1: Comparison of motif occurrences and verified binding events.

For the 216 tested transcription factors, actual binding events are on average 14-fold less frequent than binding sequence occurrences in the *A. thaliana* genome, for the respective transcription factor. The distribution on the left shows the number of individual motif occurrences for each transcription factor using the tool FIMO[1]. The distribution on the right shows the frequency of ampDAD-seq[2] verified binding events for each transcription factor. The whiskers of the boxplots are drawn up to 1.5 times the interquartile range from the respective quartile. Quartiles are drawn at the 25th and 75th percentile and the center line represents the median for each distribution.

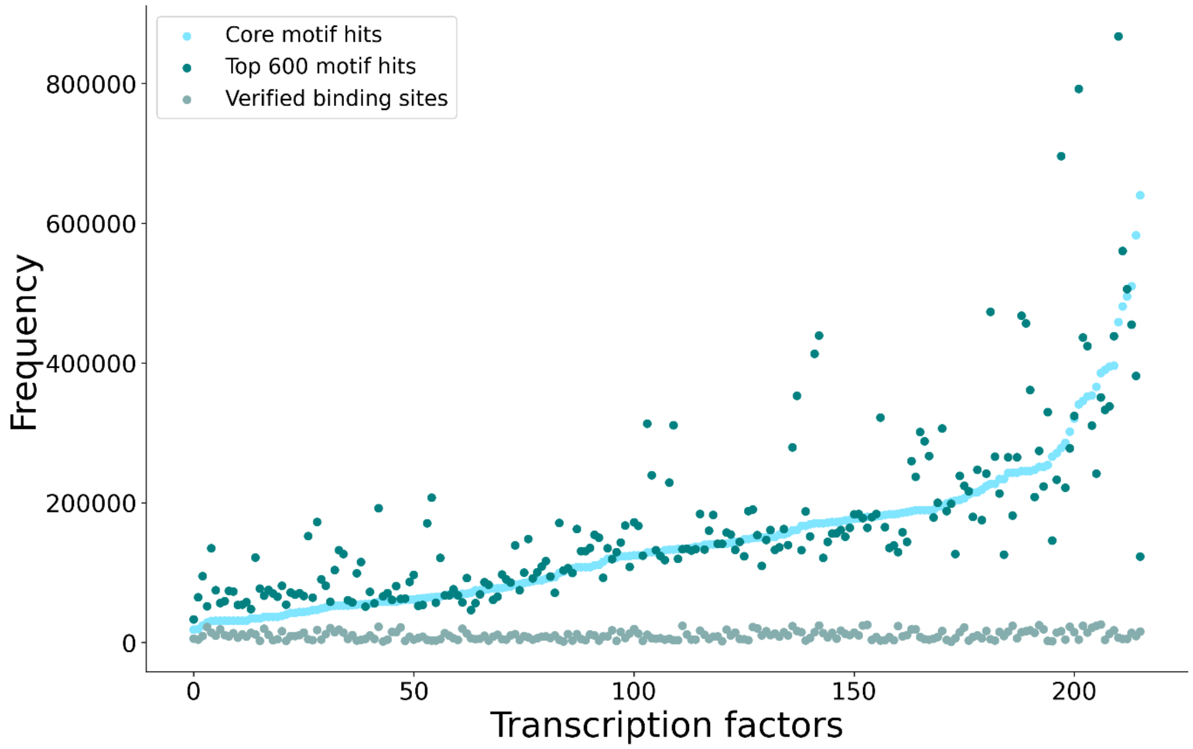

Supplementary Figure 2: Comparison between "core motif" and "top 600 peaks motif" regarding the number genomic occurrences.

Both approaches extract substantially more genomic sequences than verified binding sites for the respective transcription factor. Using the motif derived from the top 600 peaks results on average in a larger number of extracted genomic sequences that contain the motif.

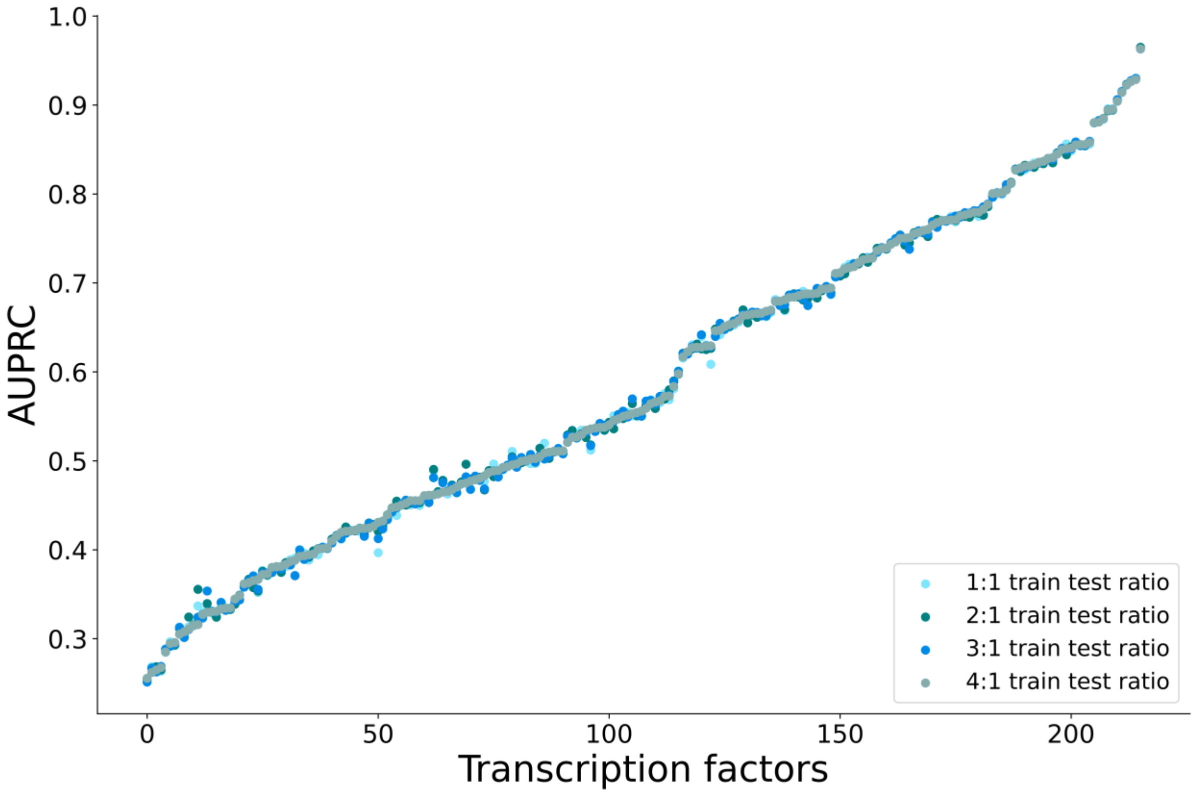

Supplementary Figure 3: Comparison of different train/test set ratios.

For each of the 216 transcription factors, four models were trained with varying ratios of train and test set size to analyse the influence of dataset size on model performance. Independent of the train/test ratio used for hyperparameter tuning, the validation set AUPRC of the random forest models was similar for almost every transcription factor.

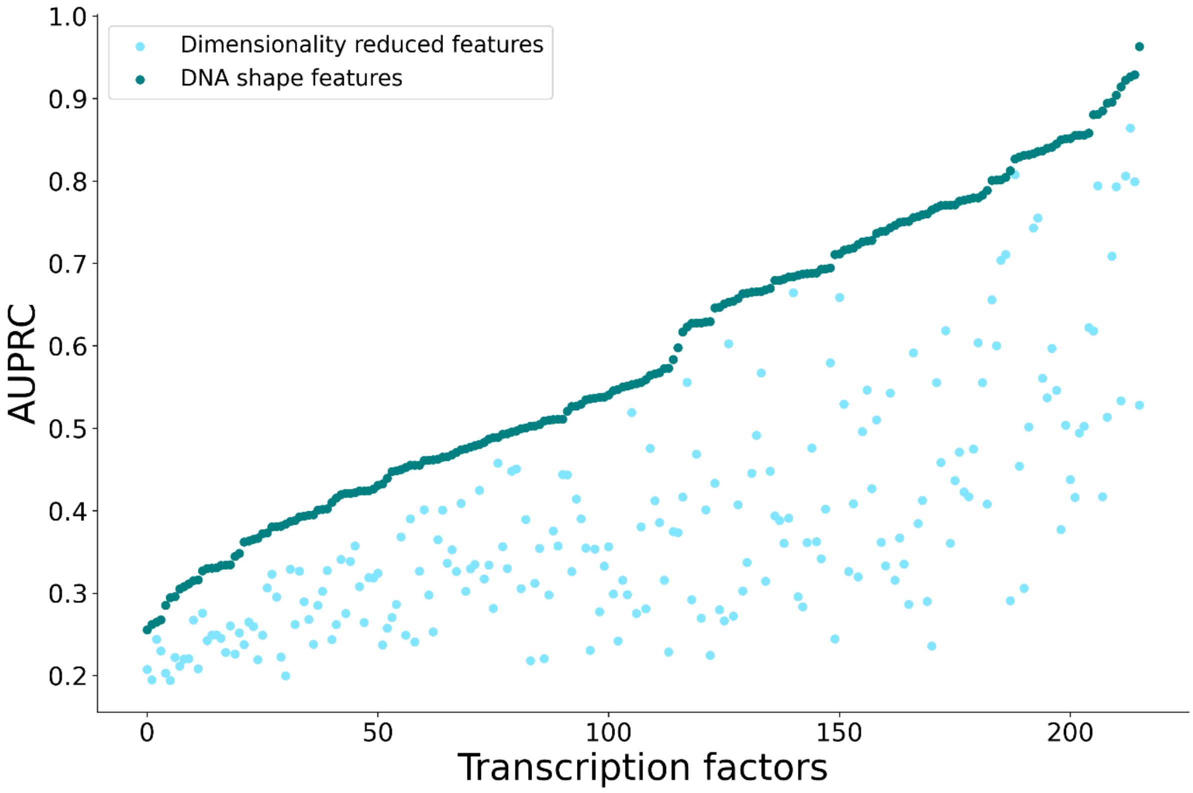

Supplementary Figure 4: Evaluation of impact on performance when dimensionality reduction is applied on the feature set.

All models were trained on the dimensionally reduced feature set and on the originally processed DNA shape feature set. After performing PCA, values of the 1st to 10th principal component were used as feature set. Training on the dimensionally reduced feature set leads to substantially lower prediction performance for most transcription factors.

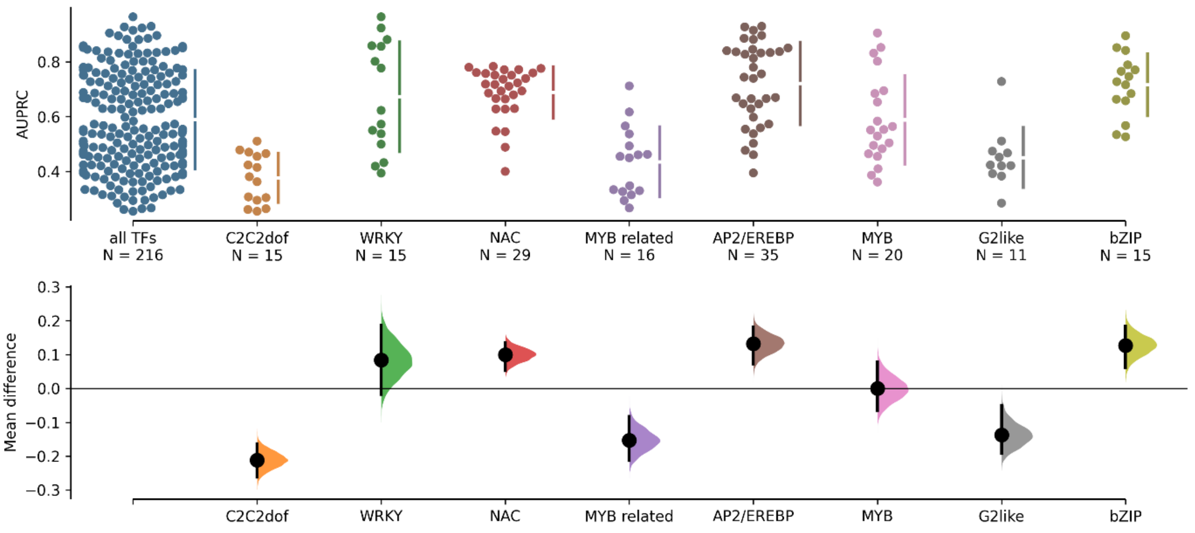

Supplementary Figure 5: Comparative analysis of family specific DNA-binding prediction performance.

The comparison is based on the area under the precision recall curve (AUPRC) using ampDAP-seq peaks as ground truth. Only families with available data of at least 10 members were investigated to ensure a robust analysis. For the families C2C2dof, MYB related and G2like a significantly ($p < 0.05$, Wilcoxon-Mann-Whitney-Test) worse performance of the random forest model regarding precision-recall relation was observed. For the transcription factor families NAC, AP2/EREBP and bZIP the performance was significantly ($p < 0.05$, Wilcoxon-Mann-Whitney-Test) better. The visualisation as well as the statistical tests were performed using the Dabest package[3].

Supplementary Table 1: Statistical test results for the comparative protein-family analysis.

For each protein family a Wilcoxon-Mann-Whitney test was performed, calculating the two-sided p-value, using the Python package Dabest[3].

control	test	p-value
all TFs	C2C2dof	0.000011
all TFs	WRKY	0.089488
all TFs	NAC	0.003943
all TFs	MYB related	0.000998
all TFs	AP2/EREBP	0.000065
all TFs	MYB	0.930433
all TFs	G2like	0.010457
all TFs	bZIP	0.006831

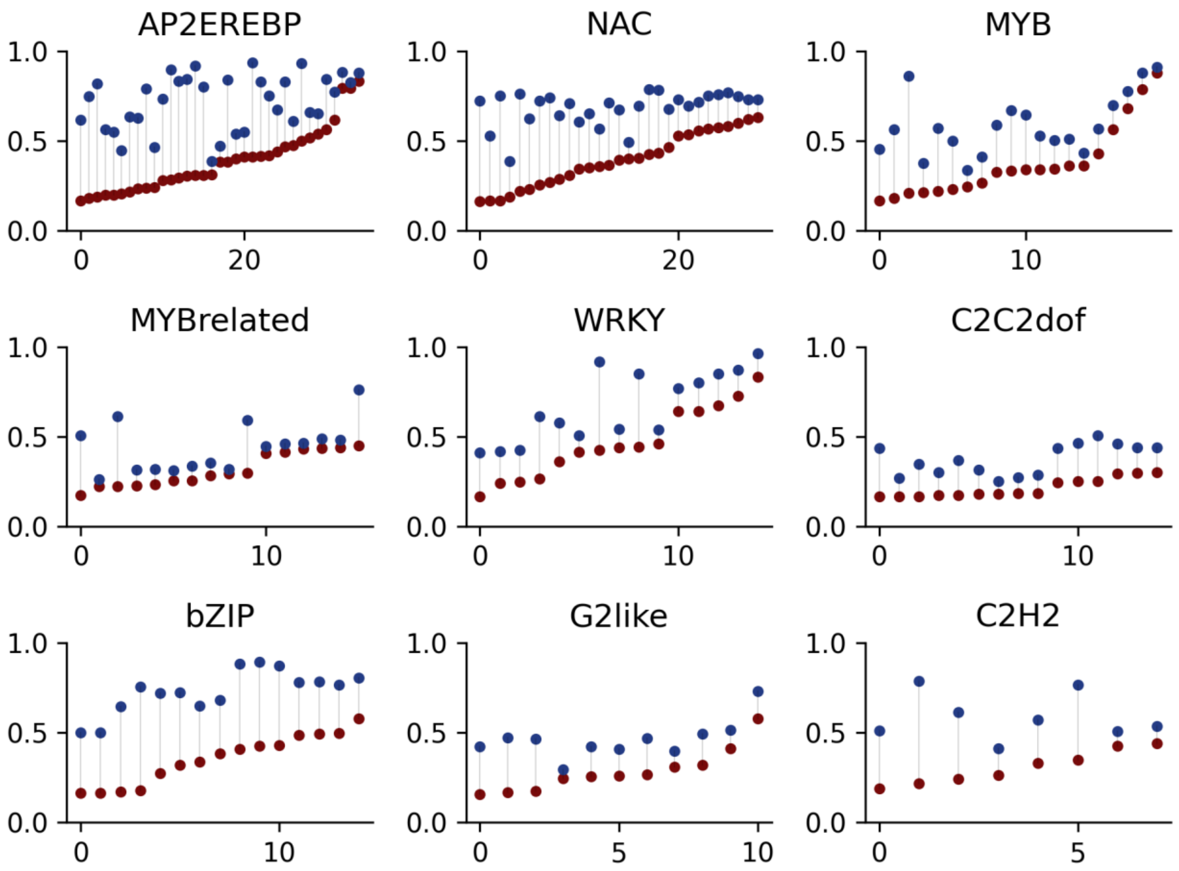

Supplementary Figure 6: Individual binding prediction improvements regarding transcription factor protein families.

Blue dots represent the area under the precision recall curve (AUPRC) using the regressor model which was trained on the DNA shape using DAP-seq data as ground truth, whereas red dots represent the AUPRC using solely the sequence motif derived from the ampDAP-seq peaks. For Members of the AP2EREBP, NAC and bZIP family, the binding prediction was consistently substantially improved. The improvement regarding the MYBrelated and C2C2dof family members was comparatively low.

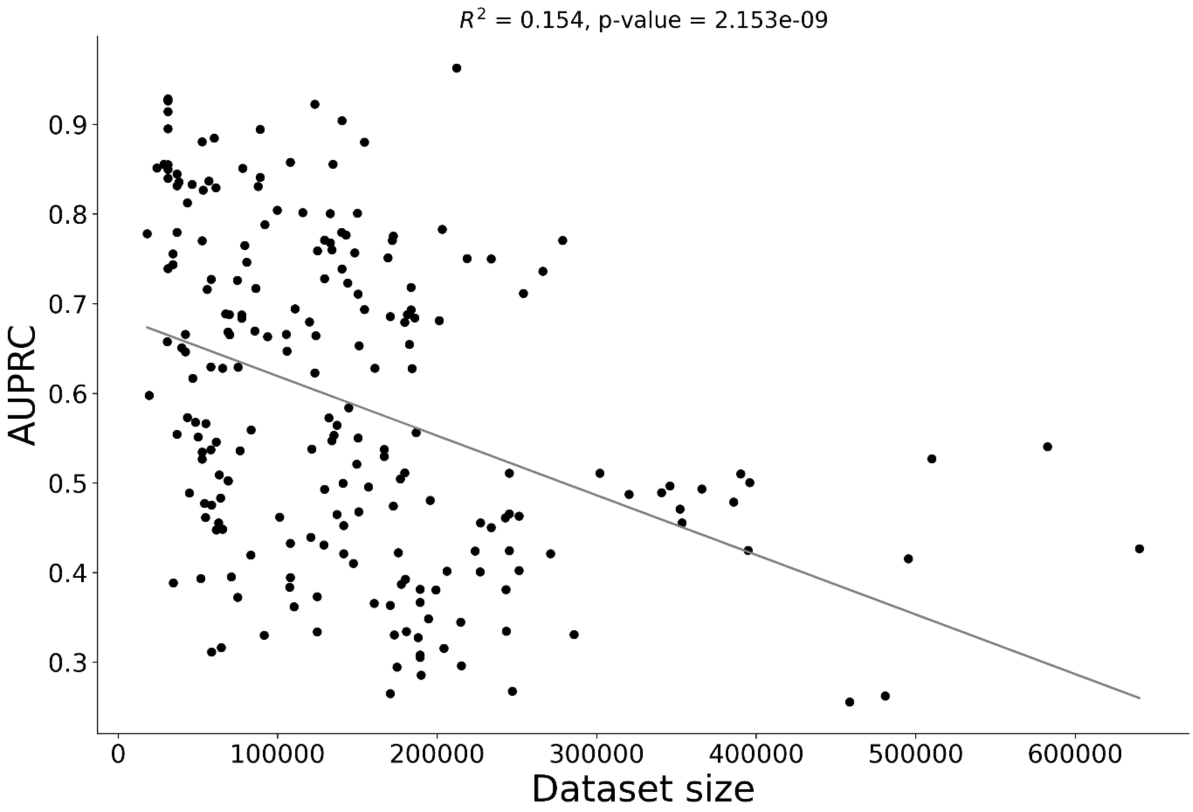

Supplementary Figure 7: Correlation between dataset size and prediction performance.

The performance of each random forest model was plotted against the number of genomic sequences containing the binding motif. Overall, performance and dataset size correlate slightly negative with a R^2 of 0.154. The linear regression, as well as the two-sided p-value, was calculated using the SciPy[4] Python package, which applies the Wald test.

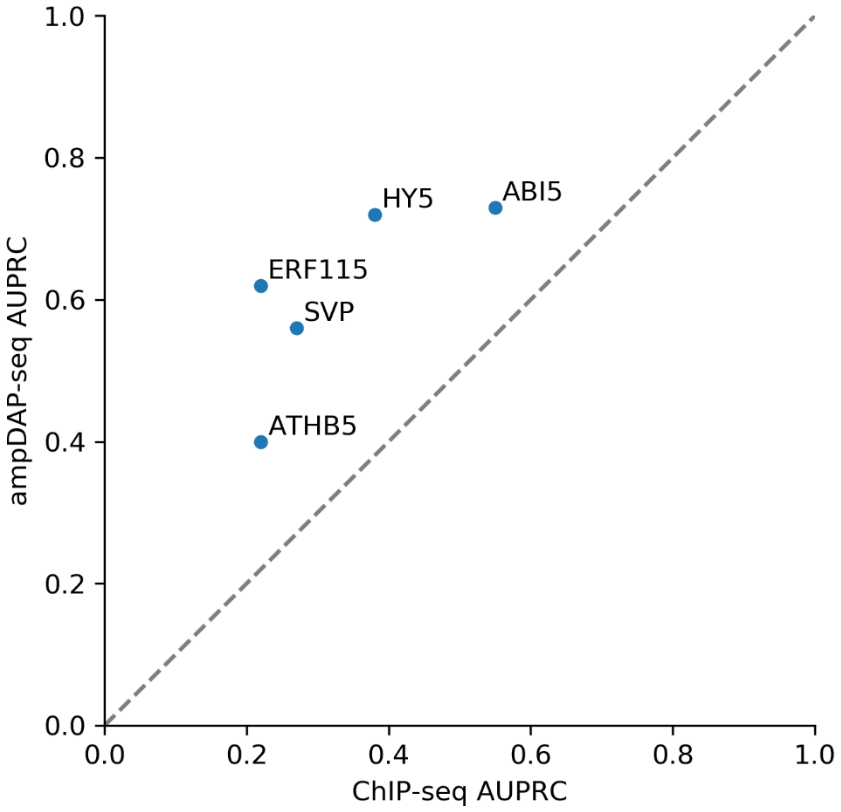

Supplementary Figure 8: Comparison of ChIP-seq and ampDAP-seq data performance.

For five transcription factors, which had data available for both experimental procedures[2,5–8], the performance of binding site inference was compared. For ChIP-seq the ChIP-seq data were used as ground truth for training and for ampDAP-seq the ampDAP-seq data were used as ground truth. The sequence motif was searched throughout the *Arabidopsis thaliana* genome to extract all putative binding sites. For each TF the random forest models trained on ampDAP-seq data had higher AUPRCs than models trained on ChIP-seq data.

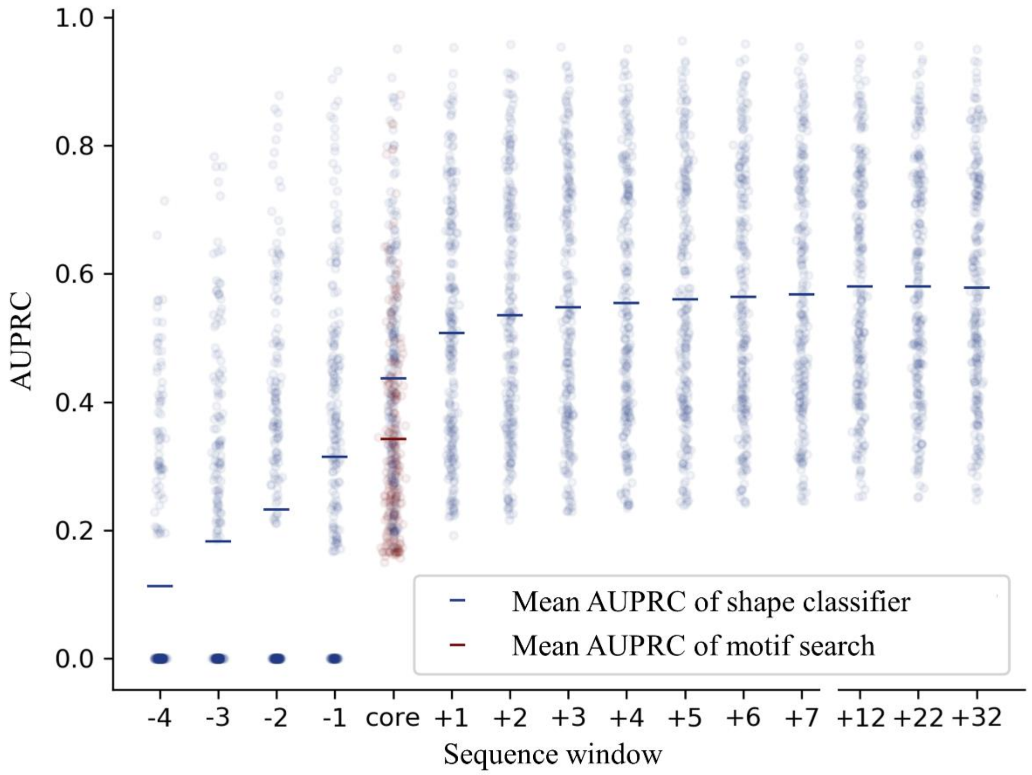

Supplementary Figure 9: Binding site prediction performance for differing sequence windows.

For each transcription factor the binding sequence window was varied up to 32 additional bases upstream and downstream from the core motif. The average AUPRC regarding the sequence search for the core motif is outperformed by calculating the DNA shape using only bases belonging to the core motif. Widening the sequence window with additional bases upstream and downstream from the core motif improves binding site predictiosn to an average AUPRC of approximately 0.6. The spread of prediction performance varies widely and is dependent on each transcription factor.

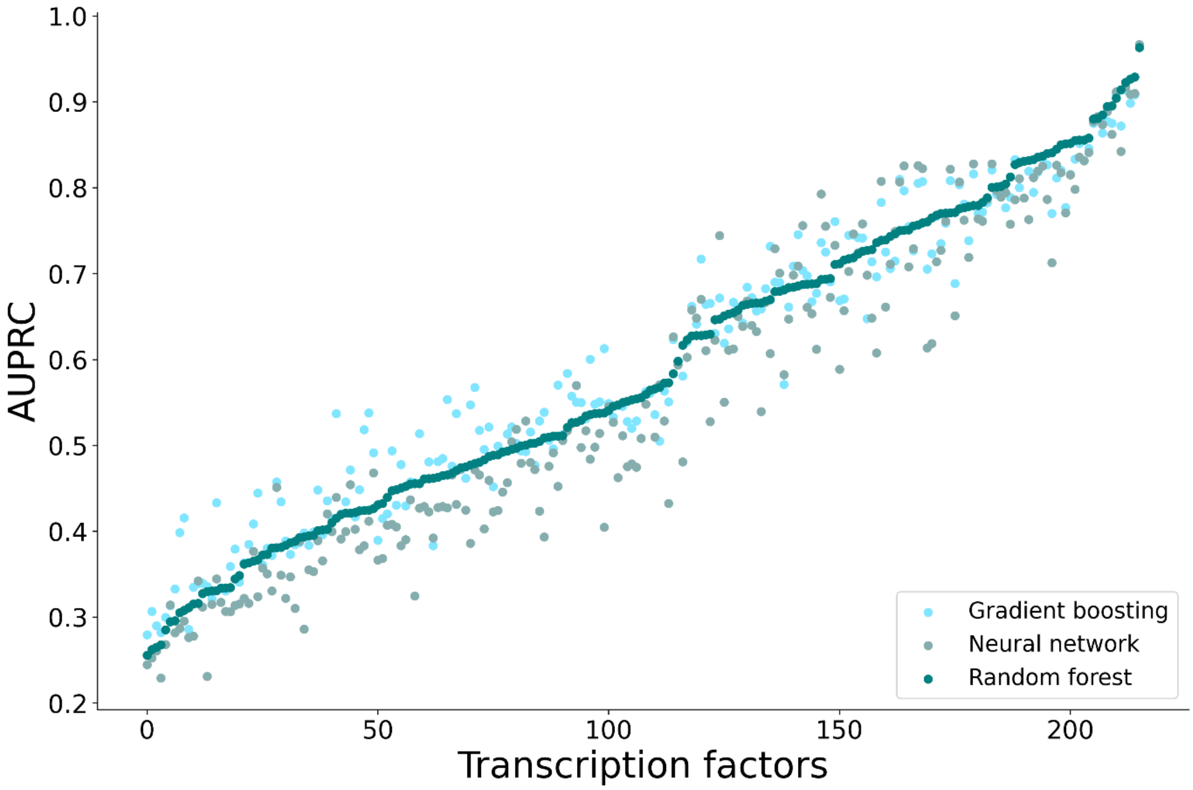

Supplementary Figure 10: Comparison of prediction performance between different machine learning approaches.

For each of the 216 transcription factors, three models using different machine learning approaches (gradient boosting, random forest and a baseline neural network) were trained. Each dot represents the performance of one of the 648 respective models. The data points are sorted according to the performance of the random forest approach.

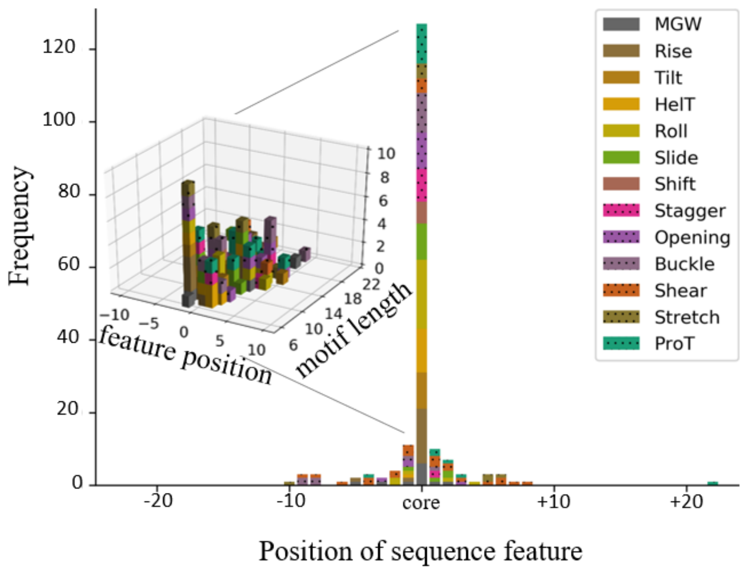

Supplementary Figure 11: Frequency of the respective top 5 most important shape features for all TFs.

For each of the 216 TFs transcription factor a random forest model was trained. The respective feature importance was extracted and all features but the top 5 most important were discarded for each TF. Most of the important shape features are located within the core motif.

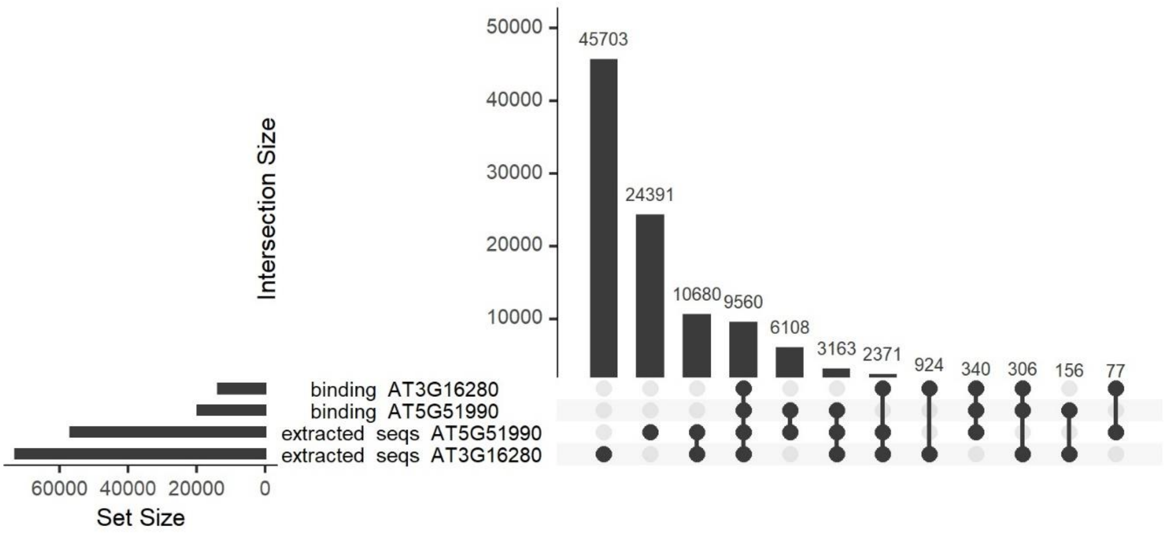

Supplementary Figure 12: Intersections of "top 600 motif" occurrences and verified bind sites for AT3G16280 and AT5G51990.

Using the published motif derived from the top 600 peaks to scan for motif occurrences results in 103,779 hits. Although, the overlap between the locations is not as strong compared to using the core motif derived from all binding events instead of the top 600. Further, a noticeable amount of verified binding sites for AT5G51990 is only found in the extracted sequences for this transcription factor when the "top 600 motif" is applied.

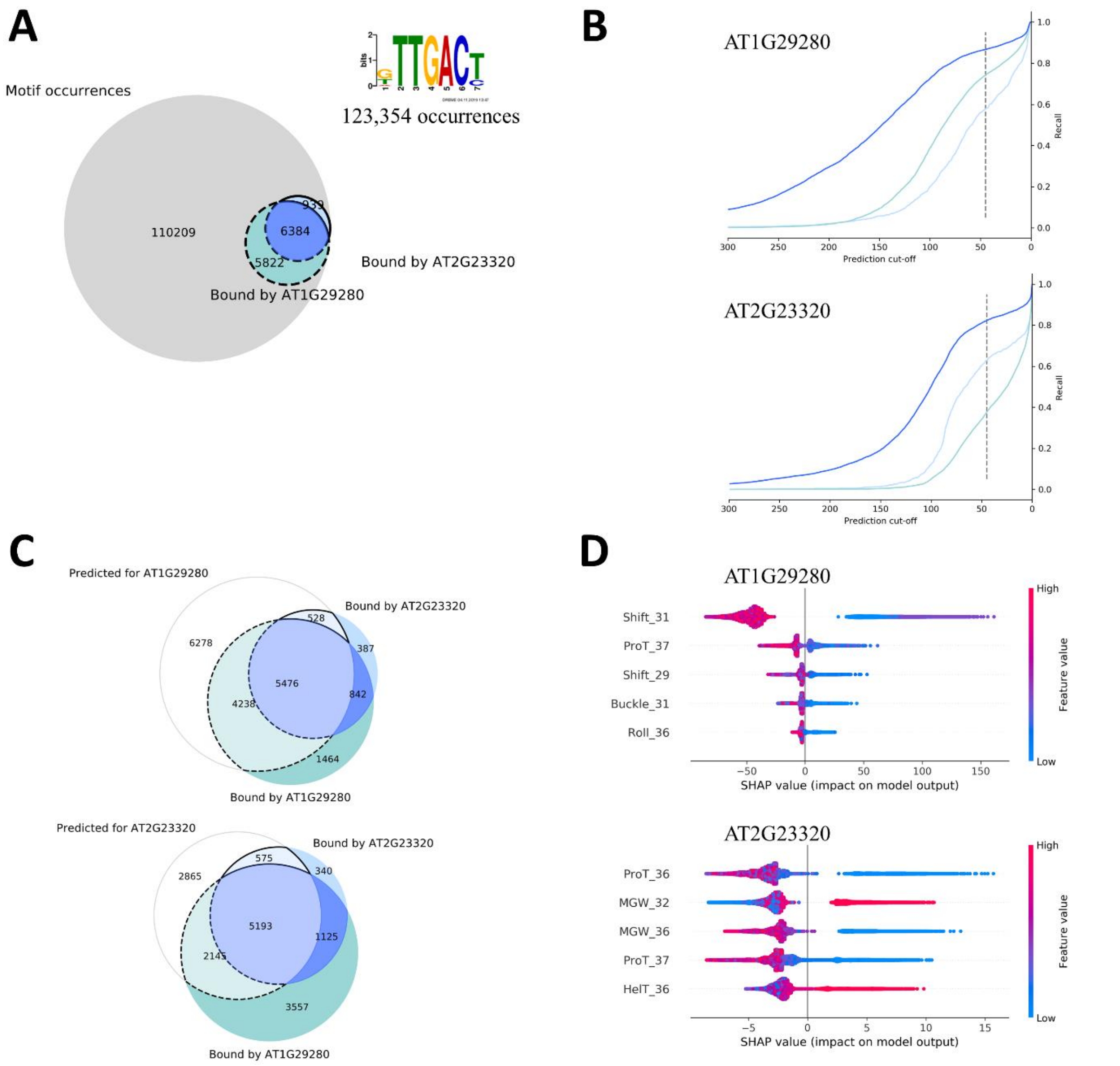

Supplementary Figure 13: Differentiation of binding specificity of two WRKY transcription factors with the same binding motif.

A) Occurrence of the TTGAC(T/C) binding motif in the *A. thaliana* genome sequence and the experimentally validated binding sequences of the WRKY TFs AT2G23320 and AT1G29280. B) Performance of the random forest regressor trained on the genomic 3D shape. C) The Venn diagrams show the sequence distributions according to the cut-off represented by the dashed line, respectively. Fields with light colours show the overlap of predicted and validated binding sequences. Dark coloured fields show the quantity of sequences, which were not predicted as bound by the model regarding the shown cut-off. D) Impact of different local shape features on the prediction of the regressor model. The most influential features are at the top. Each row represents one shape feature at a single position within the sequence. The start of the core motif is at position 30.

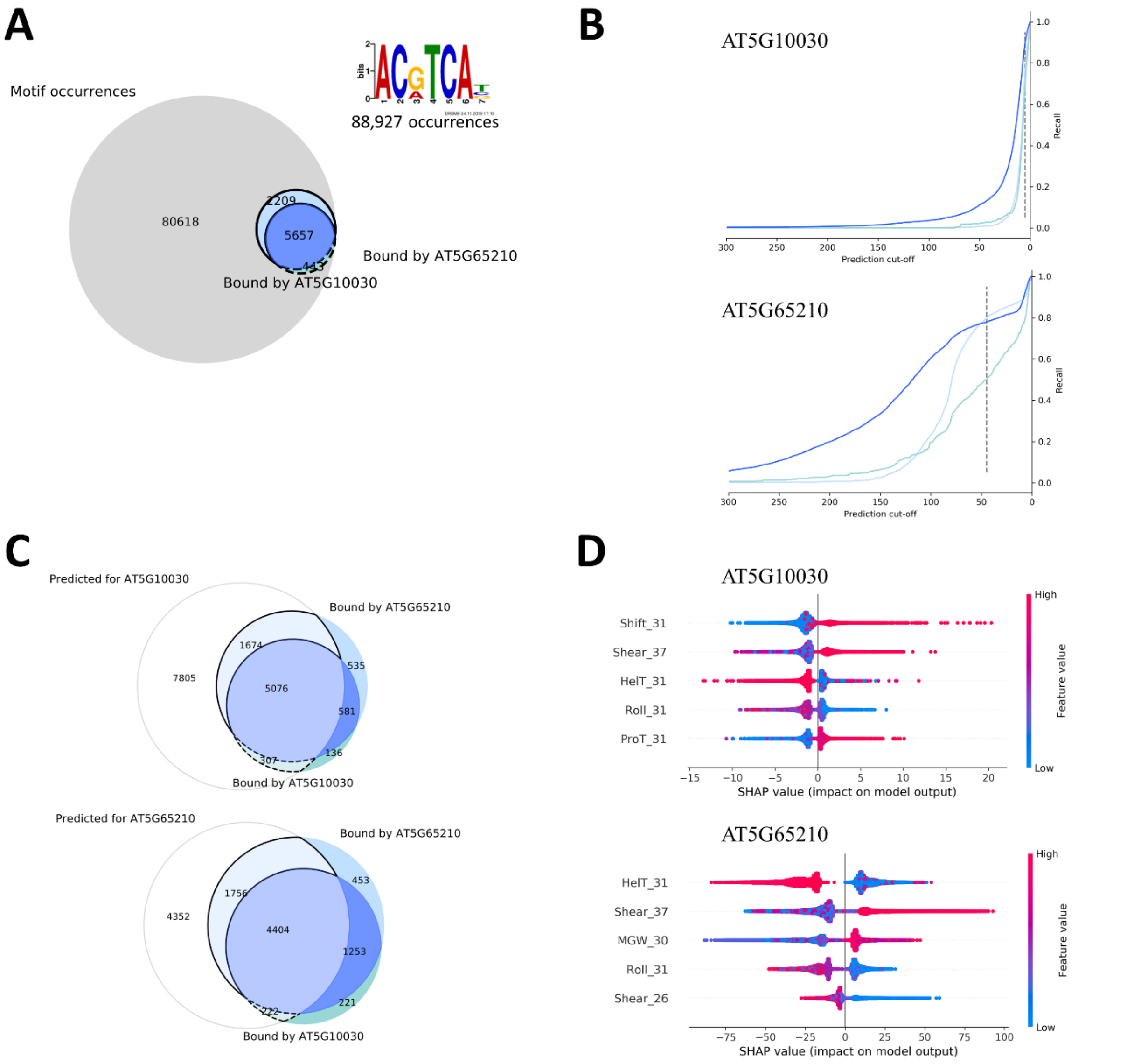

Supplementary Figure 14: Differentiation of binding specificity of two bZIP transcription factors with the same binding motif.

A) Occurrence of the ACGTCA binding motif in the *A. thaliana* genome sequence and the experimentally validated binding sequences of the bZIP TFs AT5G65210 and AT5G10030. B) Performance of the random forest regressor trained on the genomic 3D shape. C) The Venn diagrams show the sequence distributions according to the cut-off represented by the dashed line, respectively. Fields with light colours show the overlap of predicted and validated binding sequences. Dark coloured fields show the quantity of sequences, which were not predicted as bound by the model regarding the shown cut-off. D) Impact of different local shape features on the prediction of the regressor model. The most influential features are at the top. Each row represents one shape feature at a single position within the sequence. The start of the core motif is at position 30.

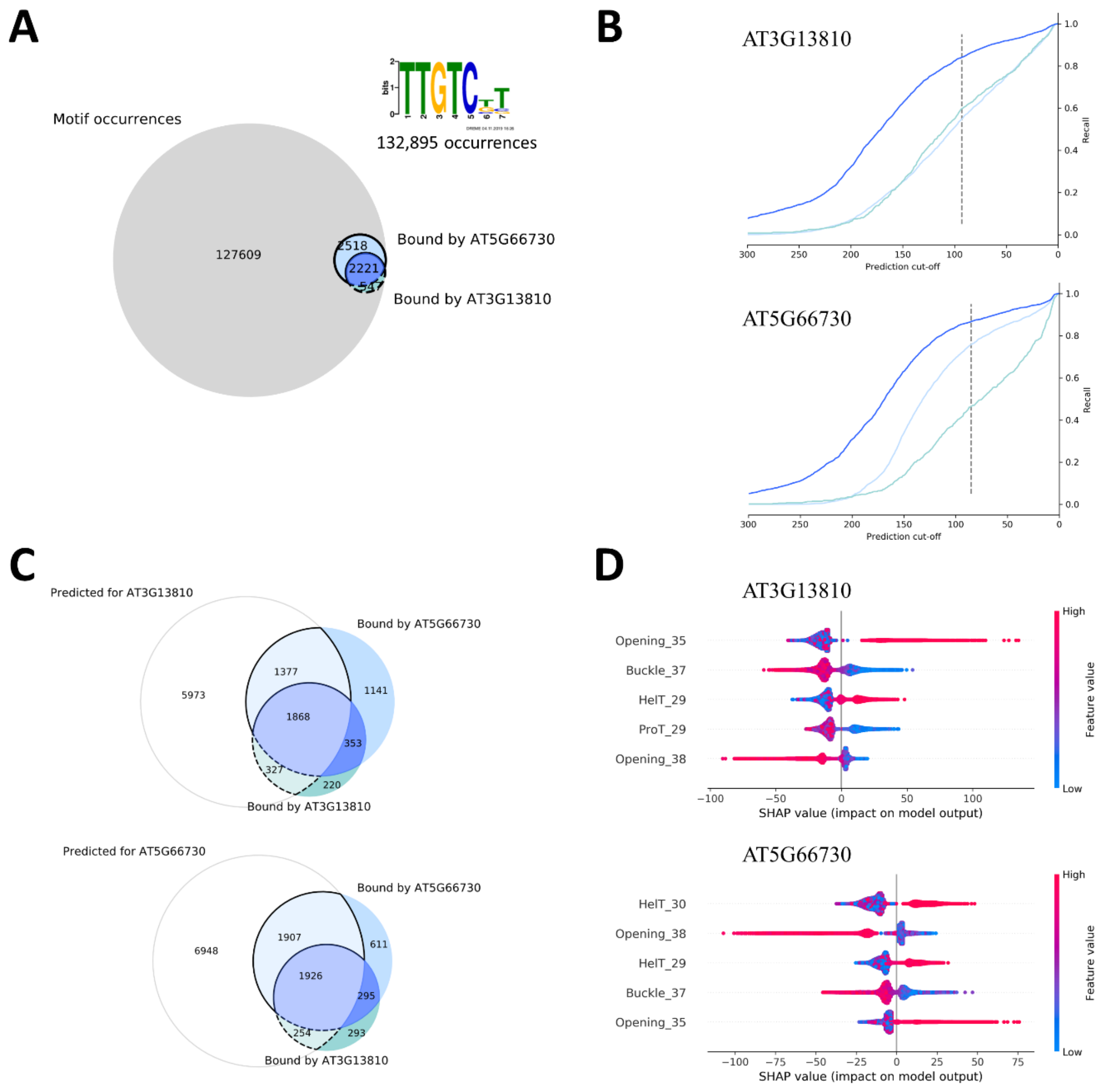

Supplementary Figure 15: Differentiation of binding specificity of two C2H2 transcription factors with the same binding motif.

A) Occurrence of the TTGTC(T/G)T binding motif in the *A. thaliana* genome sequence and the experimentally validated binding sequences of the C2H2 TFs AT5G66730 and AT3G13810. B) Performance of the random forest regressor trained on the genomic 3D shape. C) The Venn diagrams show the sequence distributions according to the cut-off represented by the dashed line, respectively. Fields with light colours show the overlap of predicted and validated binding sequences. Dark coloured fields show the quantity of sequences, which were not predicted as bound by the model regarding the shown cut-off. D) Impact of different local shape features on the prediction of the regressor model. The most influential features are at the top. Each row represents one shape feature at a single position within the sequence. The start of the core motif is at position 30.

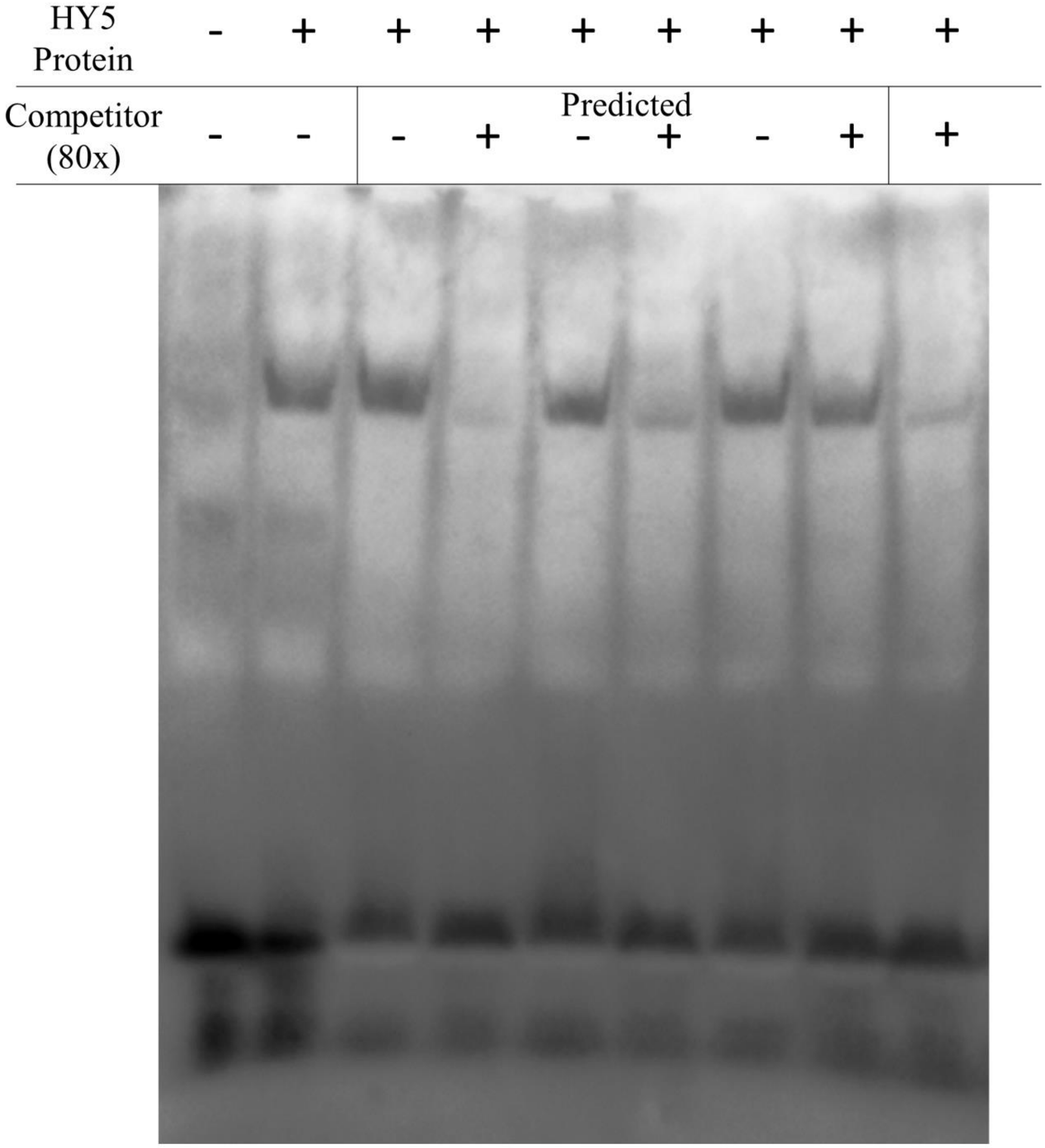

Supplementary Figure 16: Competition EMSA with the *A. thaliana* transcription factor HY5.

For experimental validation of the regressor predictions, DNA sequence with a high and low regressor prediction containing the same sequence motif not present in the genome of *A. thaliana* were generated. One sequence with a high prediction was labeled with biotin to detect DNA binding of HY5 by performing an EMSA. To compare binding affinities three sequences with high and low regressor predictions were used as competitors for the labeled sequence containing the same sequence motif. A shifted band is visible for all samples with low binding affinity prediction, one sample with high affinity prediction and the control without competitor sequence. Less visible shifted bands are observed in two samples with high predicted affinity and the control with the same competitor sequence. This EMSA experiment was performed two times. A cropped version of this gel image is visible in figure 3 and an uncropped version is available in the source data file.

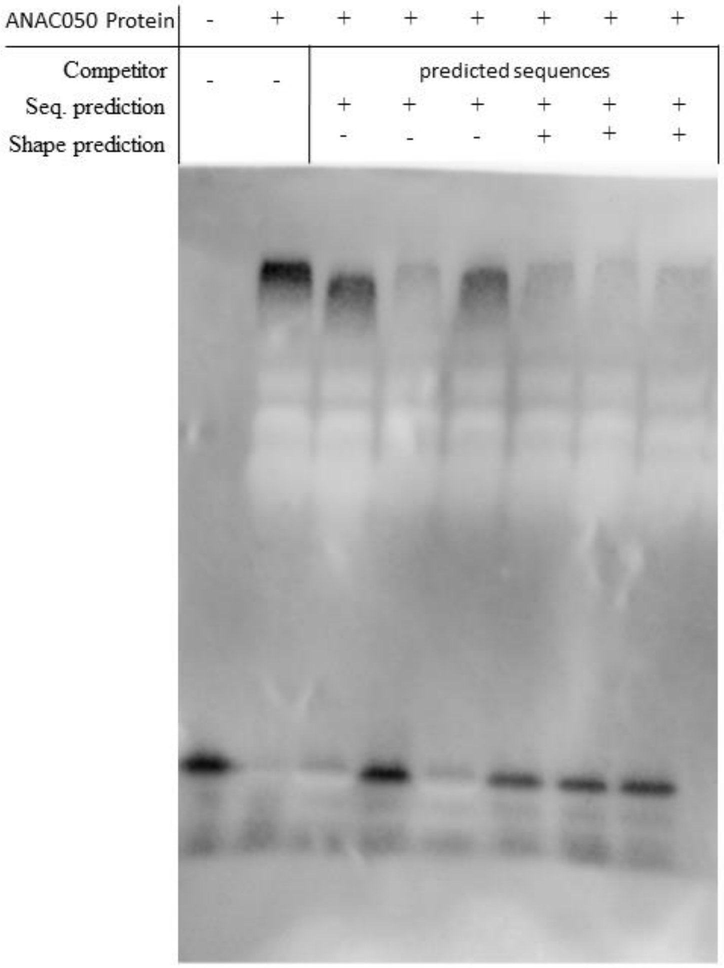

Supplementary Figure 17: Competition EMSA with the *A. thaliana* transcription factor ANAC050.

For experimental validation of the regressor predictions, DNA sequences not present in the genome of *A. thaliana* were generated. All sequences contain the extracted sequence motif for ANAC050. One sequence with a high regressor prediction was labeled with biotin to detect DNA binding of ANAC050 by performing an EMSA. To compare binding affinities three sequences with high and low regressor predictions were used as competitors for the labeled sequence. A shifted band is visible for two out of three samples with low binding affinity prediction and the control without competitor sequence. Less visible shifted bands are observed in all samples with high predicted affinity. This EMSA experiment was performed once. A cropped version of this gel image is visible in figure 3 and an uncropped version is available in the source data file.

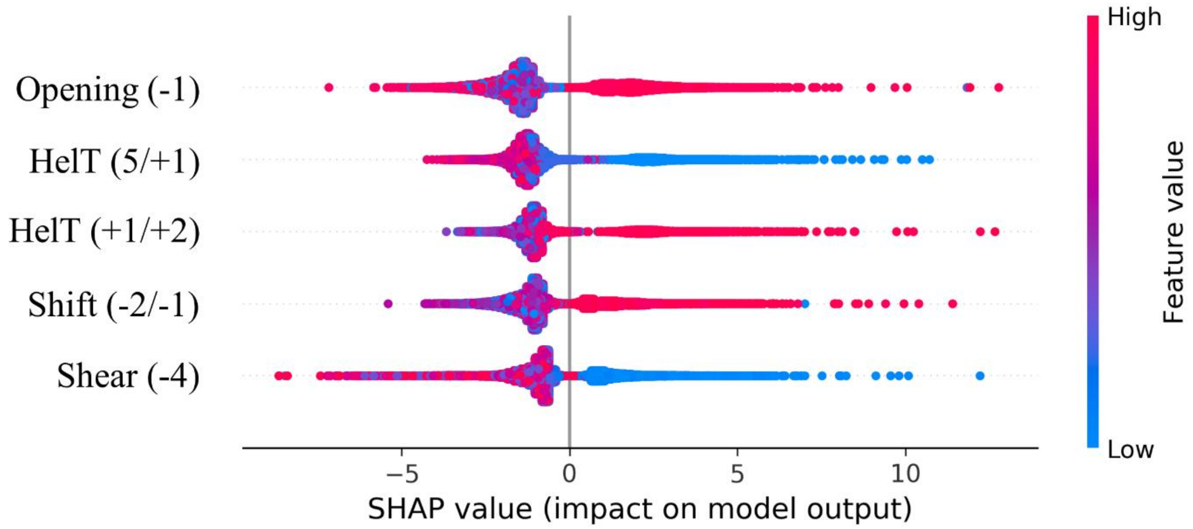

Supplementary Figure 18: Top 5 most important shape features for HY5 binding.

The SHAP Python package[9] was used to extract the most important features of the random forest model trained on experimentally validated HY5 binding sequences. The most important shape feature is the Opening at the -1 position, which is one base upstream of the motif sequence.

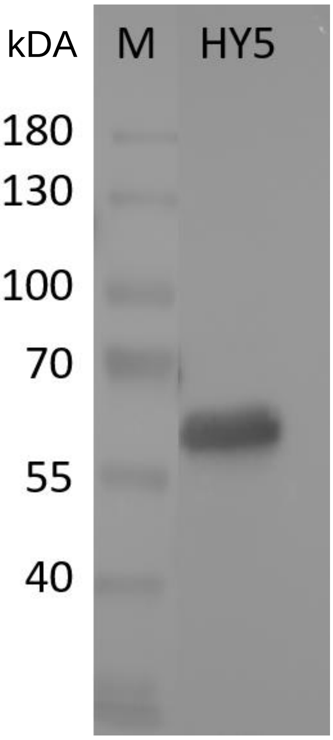

Supplementary Figure 19: Western blot of Protein-Halo-Tag fusion.

To confirm the expression of the N-terminal fusion of HY5 and the Halo tag a SDS-Page with a 10% acrylamide gel followed by a western blot on a 0,22 µm Nitrocellulose membrane (Sartorius, Göttingen, Germany) was performed. The HY5 protein was expressed with TnT® SP6 High-Yield Wheat Germ Protein Expression System (Promega, Madison, Wisconsin, United States) using 2 µg Plasmid DNA per 50 µL expression reaction (HY5). For size comparison the PageRuler™ (M) (Thermo Fisher Scientific, Waltham, Massachusetts, United States) was used. The Anti-HaloTag® Monoclonal Antibody (Promega, Madison, Wisconsin, United States) and an HRP conjugated Anti-mouse antibody (abcam, Cambridge, United Kingdom) were used to detect the fusion protein. Detection was performed using Pierce™ ECL Western Blotting Substrate (Thermo Fisher Scientific, Waltham, Massachusetts, United States) and the imaging system Fusion Fx7(Vilber, Collégien, France). The expression reaction with the plasmid DNA shows a strong band with a size of approximately 60 kDa which is consistent with the expectations, as the Halo Tag adds 33 kDA to the protein. The western blot was performed once and an uncropped version of this image is available in the source data file.

References

1. Grant, C. E., Bailey, T. L. & Noble, W. S. FIMO: scanning for occurrences of a given motif. *Bioinformatics* **27**, 1017–1018 (2011).
2. O'Malley, R. C. *et al.* Cistrome and Epicistrome Features Shape the Regulatory DNA Landscape. *Cell* **165**, 1280–1292 (2016).
3. Ho, J., Tumkaya, T., Aryal, S., Choi, H. & Claridge-Chang, A. Moving beyond P values: data analysis with estimation graphics. *Nat Methods* **16**, 565–566 (2019).
4. Virtanen, P. *et al.* SciPy 1.0: fundamental algorithms for scientific computing in Python. *Nat Methods* **17**, 261–272 (2020).
5. Song, L. *et al.* A transcription factor hierarchy defines an environmental stress response network. *Science* **354**, aag1550–aag1550 (2016).
6. Heyman, J. *et al.* ERF115 Controls Root Quiescent Center Cell Division and Stem Cell Replenishment. *Science* **342**, 860–863 (2013).
7. Burko, Y. *et al.* Chimeric Activators and Repressors Define HY5 Activity and Reveal a Light-Regulated Feedback Mechanism. *Plant Cell* **32**, 967–983 (2020).
8. Gregis, V. *et al.* Identification of pathways directly regulated by SHORT VEGETATIVE PHASE during vegetative and reproductive development in Arabidopsis. *Genome Biol* **14**, R56 (2013).
9. Lundberg, S. M. *et al.* From local explanations to global understanding with explainable AI for trees. *Nat Mach Intell* **2**, 56–67 (2020).
