# Supplementary material for: Local DNA shape is a general principle of transcription factor binding specificity in Arabidopsis thaliana
Source: Nat Commun. 2021 Nov 12;12:6549. doi: 10.1038/s41467-021-26819-2 (PMC8590021; doi:10.1038/s41467-021-26819-2)
Supplement: Supplementary file 3 — Reporting summary. [file 41467_2021_26819_MOESM3_ESM.pdf]

nature portfolio

Corresponding author(s): DBPR NCOMMS-21-09204-T

Last updated by author(s): Oct 11, 2021

Reporting Summary

Nature Portfolio wishes to improve the reproducibility of the work that we publish. This form provides structure for consistency and transparency in reporting. For further information on Nature Portfolio policies, see our Editorial Policies and the Editorial Policy Checklist.

Statistics

For all statistical analyses, confirm that the following items are present in the figure legend, table legend, main text, or Methods section.

n/a	Confirmed	
☐	☒	The exact sample size (*n*) for each experimental group/condition, given as a discrete number and unit of measurement
☐	☒	A statement on whether measurements were taken from distinct samples or whether the same sample was measured repeatedly
☐	☒	The statistical test(s) used AND whether they are one- or two-sided<br>*Only common tests should be described solely by name; describe more complex techniques in the Methods section.*
☒	☐	A description of all covariates tested
☒	☐	A description of any assumptions or corrections, such as tests of normality and adjustment for multiple comparisons
☒	☐	A full description of the statistical parameters including central tendency (e.g. means) or other basic estimates (e.g. regression coefficient) AND variation (e.g. standard deviation) or associated estimates of uncertainty (e.g. confidence intervals)
☐	☒	For null hypothesis testing, the test statistic (e.g. F, t, r) with confidence intervals, effect sizes, degrees of freedom and P value noted<br>*Give P values as exact values whenever suitable.*
☒	☐	For Bayesian analysis, information on the choice of priors and Markov chain Monte Carlo settings
☒	☐	For hierarchical and complex designs, identification of the appropriate level for tests and full reporting of outcomes
☒	☐	Estimates of effect sizes (e.g. Cohen's *d*, Pearson's *r*), indicating how they were calculated

Our web collection on statistics for biologists contains articles on many of the points above.

Software and code

Policy information about availability of computer code

Data collection	The ampDAP-seq peak calling data were obtained from the Plant Cistrome Database (neomorph.salk.edu/dap_web/pages/index.php).<br><br>All peak sequences were extracted from the A. thaliana reference genome sequence (TAIR10), obtained from https://www.arabidopsis.org/.<br><br>The query table for DNA shape translation was obtained from https://rohslab.usc.edu/DNAshape+/.
Data analysis	Data analysis required the tools FIMO and MEME-ChIP provided by the MEME-Suite (v5.0) (https://meme-suite.org/meme/). In addition, the data analysis required Python v3.7 and the Python packages pandas v1.2.4, numpy v1.19.5, scikit-learn v0.23.2, biopython v1.76, matplotlib v3.4.2, shap v0.37.0, scipy v1.7.0 and dabest v0.3.1.

For manuscripts utilizing custom algorithms or software that are central to the research but not yet described in published literature, software must be made available to editors and reviewers. We strongly encourage code deposition in a community repository (e.g. GitHub). See the Nature Portfolio guidelines for submitting code & software for further information.

Data

Policy information about availability of data

All manuscripts must include a data availability statement. This statement should provide the following information, where applicable:

- Accession codes, unique identifiers, or web links for publicly available datasets
- A description of any restrictions on data availability
- For clinical datasets or third party data, please ensure that the statement adheres to our policy

Source data are provided with this paper, containing used sequences for the EMSA, uncropped gel images and resulting values, which were used to create the

figures. To translate DNA sequence into shape features the publicly available query table (https://rohslab.usc.edu/DNAshape+/) was used. The ampDAP-seq peak calling data, which were used as ground truth to train the models, were obtained from the Plant Cistrome Database (neomorph.salk.edu/dap_web/pages/index.php).

Field-specific reporting

Please select the one below that is the best fit for your research. If you are not sure, read the appropriate sections before making your selection.

☒ Life sciences ☐ Behavioural & social sciences ☐ Ecological, evolutionary & environmental sciences

For a reference copy of the document with all sections, see nature.com/documents/nr-reporting-summary-flat.pdf

Life sciences study design

All studies must disclose on these points even when the disclosure is negative.

Sample size	For each of 216 transcription factors, which had publicly available ampDAP-seq data, models were trained. In total, 31606358 sequences were used for training and validation.
Data exclusions	Only binding events with a fraction of reads in peaks (FRiP) value > 5% were considered as ground truth for portein-DNA binding.
Replication	For model creation, as well as for splitting train and validation data, a random state was declared to ensure reproducibility.
Randomization	Sequences for each transcription factor were split into train/test and validation randomly.
Blinding	Due to the nature of the experimental setup blinding was not applicable

Reporting for specific materials, systems and methods

We require information from authors about some types of materials, experimental systems and methods used in many studies. Here, indicate whether each material, system or method listed is relevant to your study. If you are not sure if a list item applies to your research, read the appropriate section before selecting a response.

Materials & experimental systems

n/a	Involved in the study	
☐	☒	Antibodies
☒	☐	Eukaryotic cell lines
☒	☐	Palaeontology and archaeology
☒	☐	Animals and other organisms
☒	☐	Human research participants
☒	☐	Clinical data
☒	☐	Dual use research of concern

Methods

n/a	Involved in the study	
☒	☐	ChIP-seq
☒	☐	Flow cytometry
☒	☐	MRI-based neuroimaging

Antibodies

Antibodies used	Anti-HaloTag Monoclonal Antibody - Dilution 1:10000 - CatNum: G921A - Batch: 0000341144,<br>Goat Anti-Mouse IgG H&L (HRP) - Dilution 1:7000 - CatNum: ab6789 - Batch: GR3299987-2,<br>HRP Streptavidin - Dilution 1:5000 - CatNum: 405210 - Batch: B293545
Validation	Anti-HaloTag Monoclonal Antibody - https://www.promega.de/products/protein-detection/primary-and-secondary-antibodies/anti-halotag-monoclonal-antibody/?catNum=G9211,<br>Goat Anti-Mouse IgG H&L (HRP) - https://www.abcam.com/goat-mouse-igg-hl-hrp-ab6789.html,<br>HRP Streptavidin - https://www.biolegend.com/en-us/products/hrp-streptavidin-1474?GroupID=GROUP23
